# Supplementary material for: Identification of the KNOX Gene Family in Salvia miltiorrhiza Revealing Its Response Characteristics to Salt Stress
Source: Plants (Basel). 2025 Jan 24;14(3):348. doi: 10.3390/plants14030348 (PMC11821246; doi:10.3390/plants14030348)
Supplement: Supplementary file 1 [file plants-14-00348-s001.zip › plants-3405130-supplementary.pdf]

Supplementary Tables and Figures

Identification of the KNOX gene family in *Salvia miltiorrhiza* revealing its response characteristics to salt stress

Siqi Deng [1#], Wenjing Ma [1#], Chunxu Cui [1#], Shiqian Wang[1], Mei Jiang[1,2*]

[1] Key Laboratory for Natural Active Pharmaceutical Constituents Research in Universities of Shandong Province, School of Pharmaceutical Sciences, Qilu University of Technology (Shandong Academy of Sciences), Jinan, 250014, China
[2] Shandong Engineering Research Center for Innovation and Application of General Technology for Separation of Natural Products, Shandong Analysis and Test Center, Qilu University of Technology (Shandong Academy of Sciences), Jinan, 250014, China
[#] These authors contributed equally to this work.
[*] Correspondence: mjiang@qlu.edu.cn

Table S1. The results of homologous sequence alignment.

ID	query	subject	percentage	length	start in query	end in query	start in subject	end in subject	E-value
SmKNOX1	SMil_00022268	P46639	57.651	281	86	355	101	379	8.67E-96
SmKNOX2	SMil_00004618	P46639	79.104	268	91	355	131	398	9.41E-150
SmKNOX3	SMil_00019517	P46639	64.615	325	37	350	78	398	3.79E-138
SmKNOX4	SMil_00007304	F4JWP8	73.698	384	34	383	44	419	0
SmKNOX5	SMil_00013472	P46640	49.362	235	27	242	68	300	1.36E-77
SmKNOX6	SMil_00002473	P46640	50.685	292	1	268	1	270	3.23E-82
SmKNOX7	SMil_00012017	P46640	56.228	281	18	272	25	295	1.37E-95
SmKNOX8	GWHTDOEA021114	P48000	78.082	292	76	361	136	423	8.7E-167
SmKNOX9	GWHTDOEA020617	P48000	76.721	305	72	374	133	431	1.5E-173
SmKNOX10	GWHTDOEA020485	P46640	55.963	327	1	314	1	308	2.1E-118

Table S2. The results of HMM search.

ID	Query	PF03789				PF03790				PF03791				PF05920			
		**E-value**	**score**	**E-value**	**score**	**E-value**	**score**	**E-value**	**score**	**E-value**	**score**	**E-value**	**score**	**E-value**	**score**	**E-value**	**score**
SmKNOX1	SMil_00022268	3.00E-11	43.7	5.20E-11	42.9	1.10E-18	67.4	1.10E-18	67.4	1.80E-21	76.1	4.40E-21	74.9	4.10E-19	68.9	2.00E-18	66.7
SmKNOX2	SMil_00004618	2.60E-10	40.6	3.70E-09	37	1.10E-22	80.2	2.10E-22	79.3	9.10E-25	86.7	2.30E-24	85.4	4.50E-19	68.7	1.30E-18	67.3
SmKNOX3	SMil_00019517	1.50E-09	38.3	3.70E-09	37	2.60E-22	79	5.00E-22	78.1	4.10E-24	84.6	4.10E-24	84.6	8.40E-19	67.9	1.80E-18	66.8
SmKNOX4	SMil_00007304	1.20E-06	29	3.10E-06	27.7	1.70E-19	70	3.20E-19	69.1	3.10E-18	65.8	3.10E-18	65.8	5.60E-19	68.4	1.30E-18	67.3
SmKNOX5	SMil_00013472	2.10E-06	28.2	3.10E-06	27.7	9.50E-20	70.8	1.60E-19	70.1	1.20E-19	70.3	2.10E-19	69.5	2.50E-18	66.4	5.90E-18	65.2
SmKNOX6	SMil_00002473	6.90E-05	23.4	0.00016	22.2	1.80E-21	76.3	3.20E-21	75.5	4.80E-21	74.8	8.20E-21	74	1.30E-05	25.7	3.60E-05	24.2
SmKNOX7	SMil_00012017	7.30E-05	23.3	0.00016	22.2	1.60E-21	76.5	2.90E-21	75.7	5.00E-21	74.7	8.50E-21	74	1.40E-18	67.2	3.70E-18	65.8
SmKNOX8	GWHTDOEA021114	0.0001	22.9	0.0001	22.9	3.2E-18	66	6.8E-18	64.9	9.8E-16	57.8	9.8E-16	57.8	1.3E-18	67.4	1.3E-18	67.4
SmKNOX9	GWHTDOEA020617	0.00012	22.7	0.00012	22.7	1.8E-18	66.8	1.8E-18	66.8	8.3E-16	58.1	8.3E-16	58.1	4.8E-19	68.7	1.3E-18	67.3
SmKNOX10	GWHTDOEA020485	0.0013	19.4	0.0031	18.2	8.3E-21	74.3	1.7E-20	73.3	3.8E-25	88	6.8E-25	87.1	1.8E-18	66.9	3.7E-18	65.9

Table S3. The prediction of SmKNOX protein core binding motifs upstream of target genes.

Gene ID	Gene	Position	Rel Position	Sequence
SMil_00000335	REVOLUTA	38	-1963	gggTGACggc
SMil_00020416	S11 MYB	1579	-422	ataTGACatg
SMil_00028207	S11 MYB	1719	-282	gatTGACcat
SMil_00028207	S11 MYB	1759	-242	caaTGACaca
SMil_00020416	S11 MYB	255	-1746	tatTGACtcg
SMil_00020416	S11 MYB	269	-1732	aggTGACaag
SMil_00020416	S11 MYB	501	-1500	gggTGACact
SMil_00020416	S11 MYB	1307	-694	aaaTGACaat
SMil_00020416	S11 MYB	1758	-243	aatTGACtca
SMil_00028207	S11 MYB	1586	-415	ttaTGACttc
SMil_00020647	S11 MYB	639	-1362	aatTGACaaa
SMil_00020647	S11 MYB	1810	-191	catTGACttc
SMil_00028207	S11 MYB	1680	-321	tagTGACata
SMil_00028207	S11 MYB	332	-1669	tctTGACgtt
SMil_00000335	REVOLUTA	586	-1415	aaaTGACata
SMil_00000335	REVOLUTA	702	-1299	gatTGACaat
SMil_00000335	REVOLUTA	806	-1195	attTGACaaa
SMil_00000335	REVOLUTA	1457	-544	tttTGACatg
SMil_00001022	REVOLUTA	236	-1765	ttaTGACaaa
SMil_00028207	S11 MYB	66	-1935	gtgTGACtta
SMil_00003437	ABI3	887	-1114	ggtTGACgtc
SMil_00003437	ABI3	1061	-940	tcaTGACatg
SMil_00003437	ABI3	1697	-304	gatTGACaac
SMil_00010684	GA20ox1	690	-1311	taaTGACctc
SMil_00010684	GA20ox1	967	-1034	ttgTGACaat
SMil_00010684	GA20ox1	1707	-294	aaaTGACaaa
SMil_00010684	GA20ox1	1737	-264	aatTGACtca

Table S4. The primer sequences of qPCR used in this project.

ID	Sequence
qPCR-SmKNOX1-F	TGGACACCACCAACGACG
qPCR-SmKNOX1-R	CGCCTCCTCTAGCTTCGC
qPCR-SmKNOX2-F	AGGAGAAGAGCGAGGGCA
qPCR-SmKNOX2-R	CGTGGATCGATCTCCGCC
qPCR-SmKNOX3-F	CAGCTGGTGGGAGTTGCA
qPCR-SmKNOX3-R	GAGGGCTTCCAATGGCGT
qPCR-SmKNOX4-F	TCGCCGGCTTCCAACAAT
qPCR-SmKNOX4-R	GCTCTCGCTTCTGCTGCT
qPCR-SmKNOX5-F	CGCATCGATCTCGCCCTT
qPCR-SmKNOX5-R	AGGCGGGATAAAGGGGGT
qPCR-SmKNOX6-F	TACGGCAGCCACATCAGC
qPCR-SmKNOX6-R	TTCCGTGGGATAGGGCCA
qPCR-SmKNOX7-F	TGGCCCTATCCCACGGAA
qPCR-SmKNOX7-R	GAGAGATTGTCCGGCGCA
qPCR-SmKNOX8-F	TTCAGCTCCCCCGCTACT
qPCR-SmKNOX8-R	GACTTTGGGTGGGGAGCC
qPCR-SmKNOX9-F	CCCCCACTACTCCGACCA
qPCR-SmKNOX9-R	TGGTGGAGGGAGGAGGTG
qPCR-SmKNOX10-F	ATCTGATGATGGCGGCGG
qPCR-SmKNOX10-R	CGCCGCGAACGTTAATGG
SM-β-actin-F	AGGAACCACCGATCCAGACA
SM-β-actin-R	GGTGCCCTGAGGTCCTGTT
REVOLUTA_1_F	GCGCCACATCTCGTACGA
REVOLUTA_1_R	TGACAAGGCGAGGCAGTG
REVOLUTA_2_F	GAGCGTGCCAGAGGTGTT
REVOLUTA_2_R	GCTGCCGACCAAGACCAT
ABI3_F	GCGAGCTGGCGATGATCT
ABI3_R	GGTGGACCGCTTCAGCTT
GA20ox1_F	ACCAAGACACAGTCGCCG
GA20ox1_R	CCGCTCTGTGCAAACAGC
S11 MYB_1_F	AAGGCGGTGGTGGACTTG
S11 MYB_1_R	TTCCGTGCTCCCGTTGAC
S11 MYB_2_F	CGACCCTCCTCAACTCGC
S11 MYB_2_R	GACGCTAGGGTCATCGCC
S11 MYB_3_F	GAAAGGCCCGTGGACTCC
S11 MYB_4_R	GTCCGTTCTTCCCGGCAA

Table S5. The primer sequences of gene cloning used in this project.

ID	Sequence
pHK-SmKNOX1-F	cgagctcggtacccgggatccATGGAGAGTGGCGGTGGTG
pHK-SmKNOX1-R	cgcgtacgagatctggtcgacGAGGAGCGCAGGCGATATATC
pHK-SmKNOX2-F	cgagctcggtacccgggatccATGGAAGAATACAATCTCCAGCTGA
pHK-SmKNOX2-R	cgcgtacgagatctggtcgacGGGGCCGAGCCTGTAGGG
pHK-SmKNOX3-F	cgagctcggtacccgggatccATGGAGGAGTACAATCATGAGATGAG
pHK-SmKNOX3-R	cgcgtacgagatctggtcgacGGGCCCCAATCTGTAAGGG
pHK-SmKNOX4-F	cgagctcggtacccgggatccATGGCTCTCCAGCATTTCGG
pHK-SmKNOX4-R	cgcgtacgagatctggtcgacCCTTTTGCGTTTGCTTTTCAA
pHK-SmKNOX5-F	cgagctcggtacccgggatccATGGATGAGCATCTCTACGCATC
pHK-SmKNOX5-R	cgcgtacgagatctggtcgacCAAAATAGCATCATTGAAAGAGGC
pHK-SmKNOX6-F	cgagctcggtacccgggatccATGGATGAAATGTATGGGTTTGATAC
pHK-SmKNOX6-R	cgcgtacgagatctggtcgacTGAACCAATTGTTGATCTGCTTCT
pHK-SmKNOX7-F	cgagctcggtacccgggatccATGTCACCGGAGAATATGATGATG
pHK-SmKNOX7-R	cgcgtacgagatctggtcgacGTGATCTTGGGGGACAAAATTG
pHK-SmKNOX8-F	cgagctcggtacccgggatccATGAAGGGGGTGCAGTGCA
pHK-SmKNOX8-R	cgcgtacgagatctggtcgacTTAGTTGTTGTTTTTGTCACCTGCA
pHK-SmKNOX9-F	cgagctcggtacccgggatccATGGCCTTTCAGCAGGACCA
pHK-SmKNOX9-R	cgcgtacgagatctggtcgacCGAGAAACGCTCATTAGCAGTTT
pHK-SmKNOX10-F	cgagctcggtacccgggatccATGGACGAAATGTATGGGATTCA
pHK-SmKNOX10-R	cgcgtacgagatctggtcgacATCTCCATTCATGAATAAATGTCCAG
1300GPF_F	CTATCCTTCGCAAGACCCTTC
1300GPF_R	TGAAGGTGGTCACGAGGG

SmKNOX1.dna (Circular / 1023 bp)

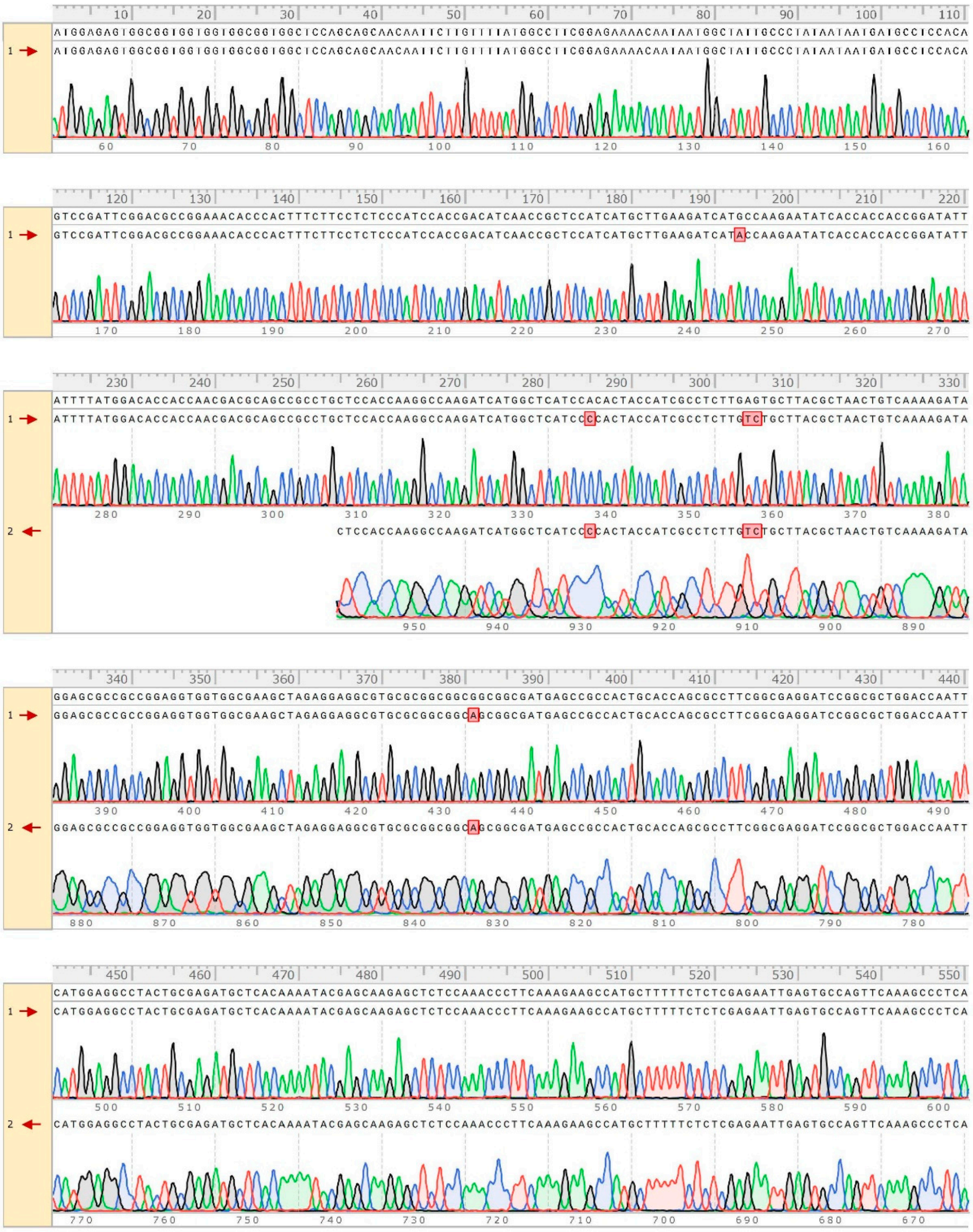

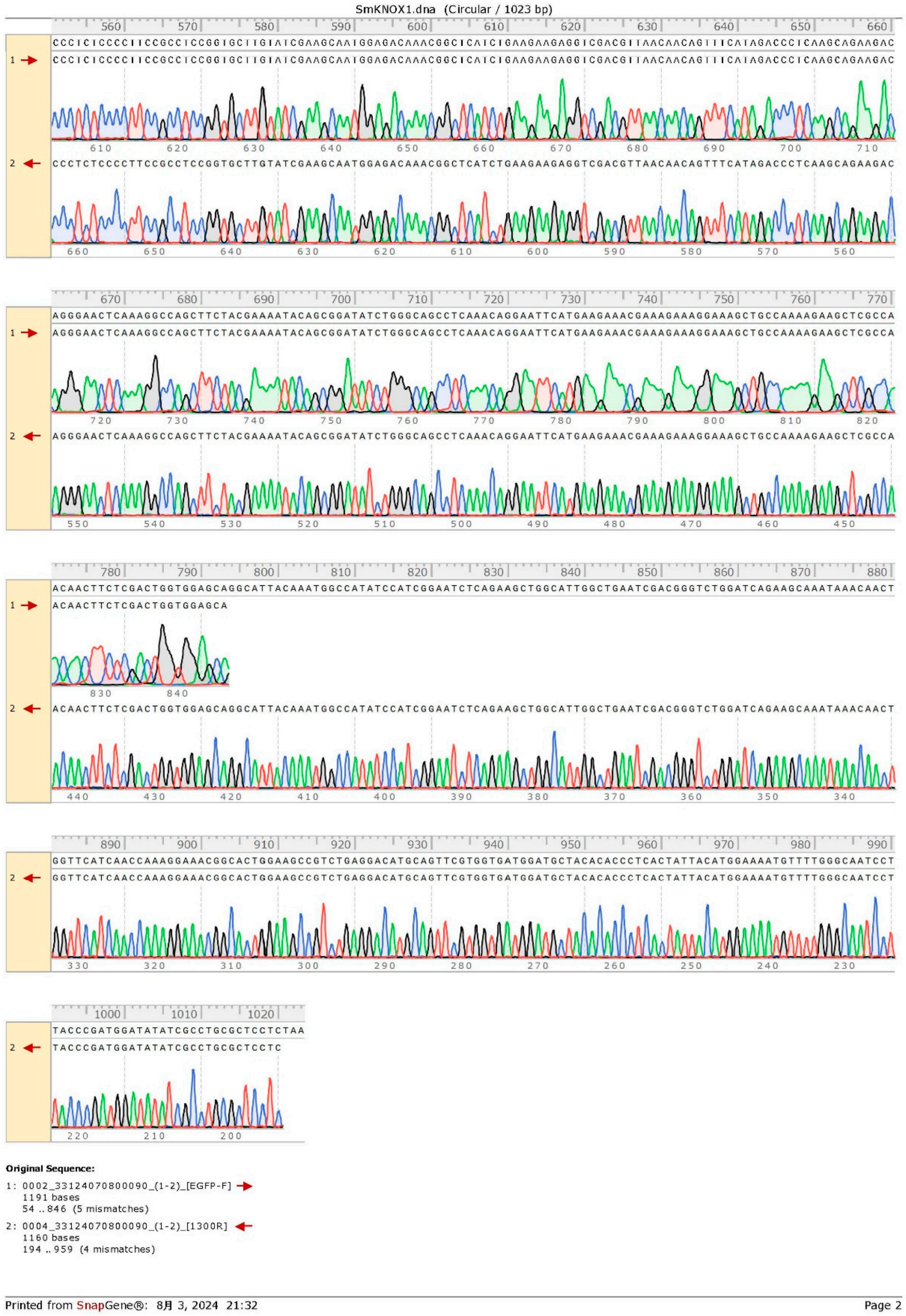

Figure S1. The gene cloning results of SmKNOX1. The base sequence above represents the sequence from reference genome, and the numbers above it represent the base positions. The base sequence below represents the Sanger sequencing result of gene cloning, and the bottom represents the chromatogram of Sanger sequencing.

SmKNOX2.dna (Linear / 1068 bp)

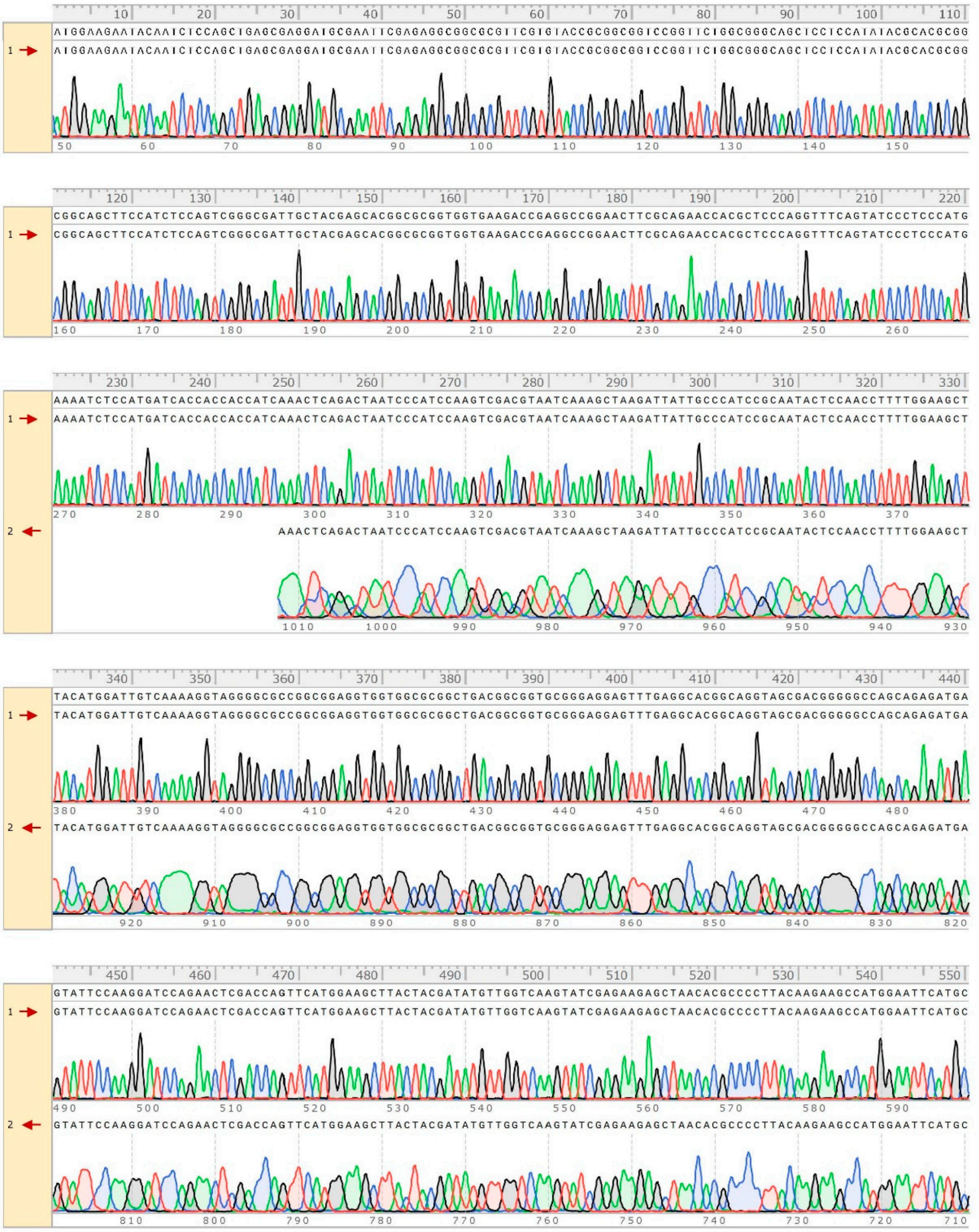

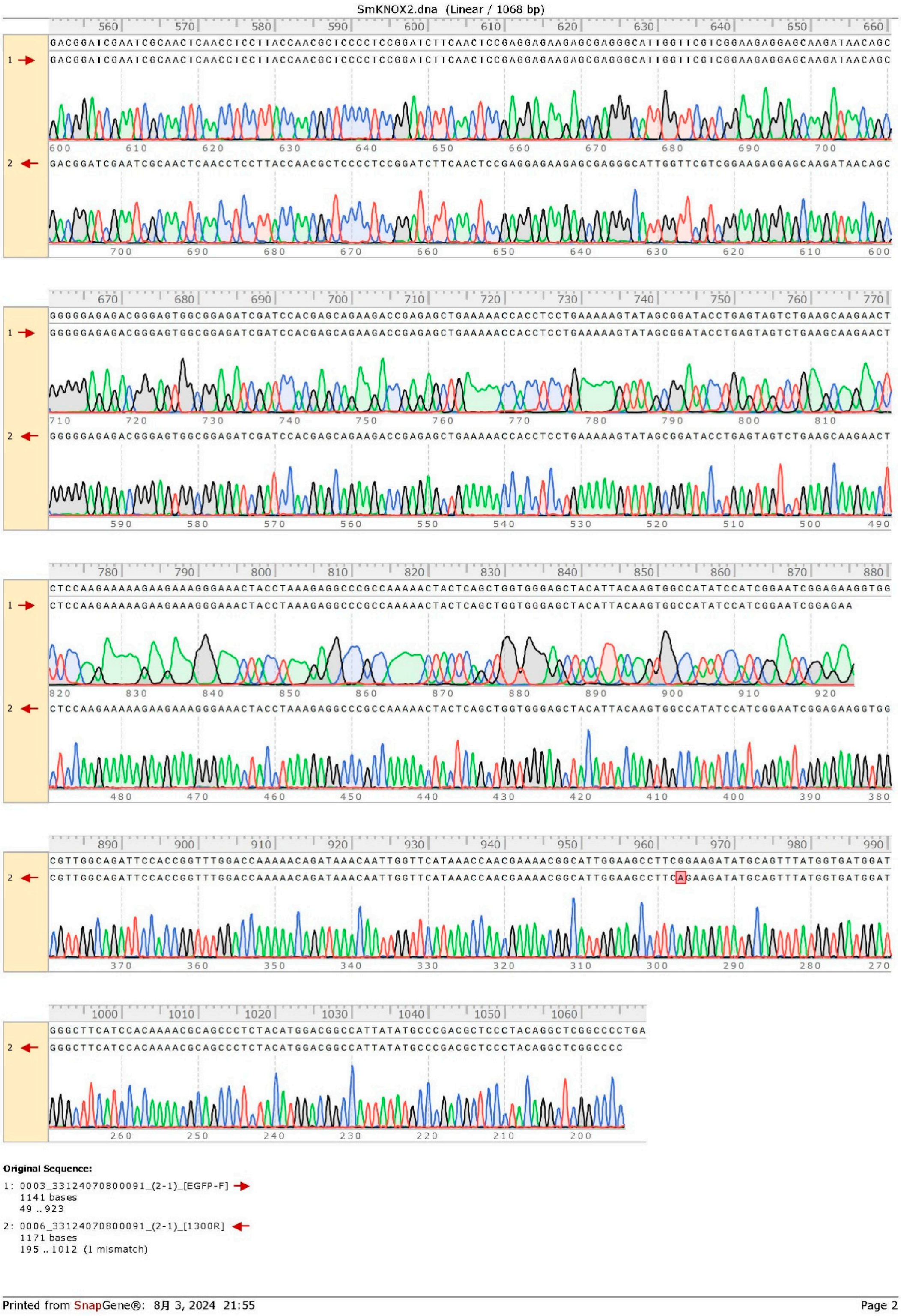

Figure S2. The gene cloning results of SmKNOX2. The base sequence above represents the sequence from reference genome, and the numbers above it represent the base positions. The base sequence below represents the Sanger sequencing result of gene cloning, and the bottom represents the chromatogram of Sanger sequencing.

SmKNOX3.dna (Linear / 1053 bp)

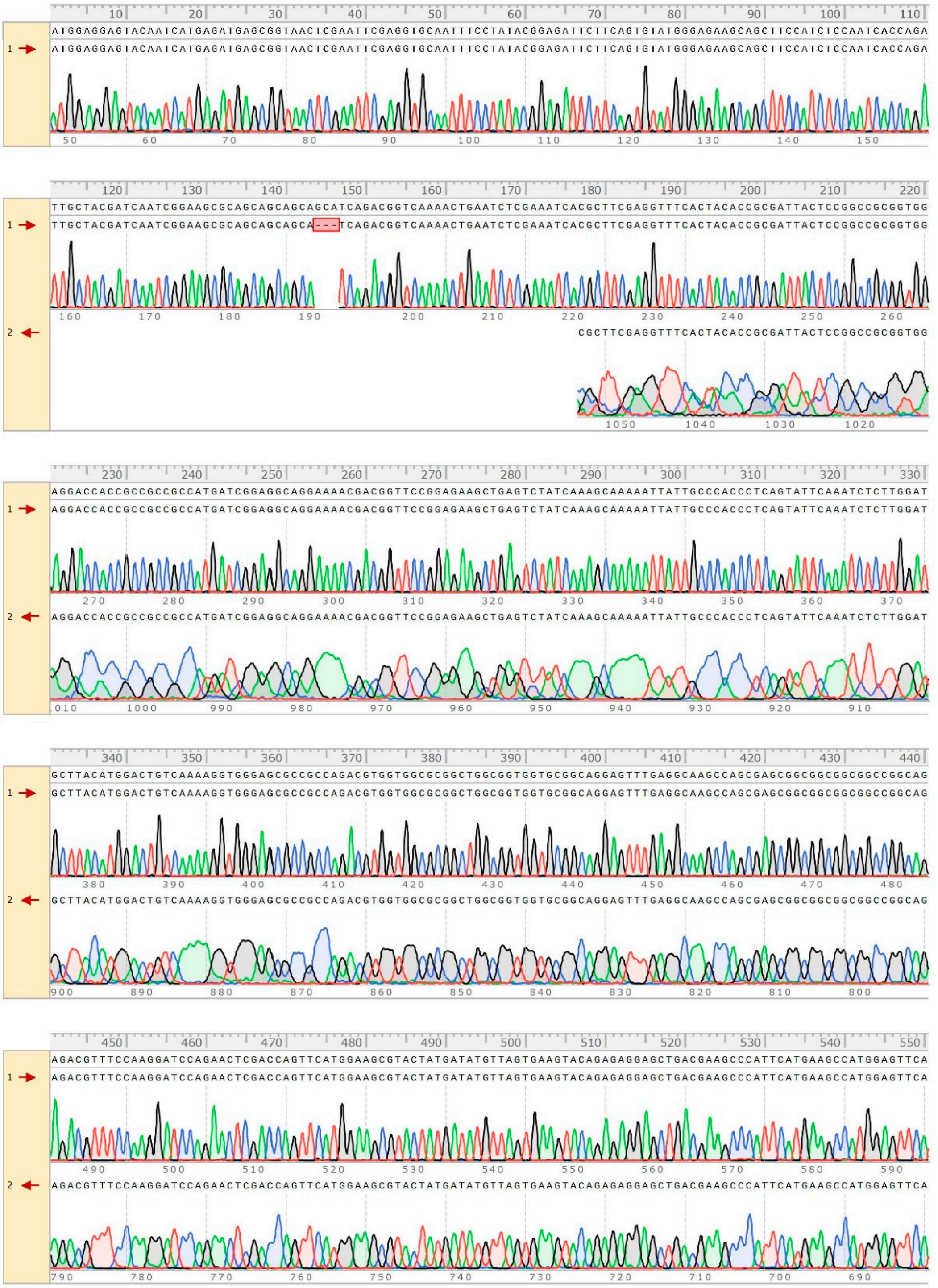

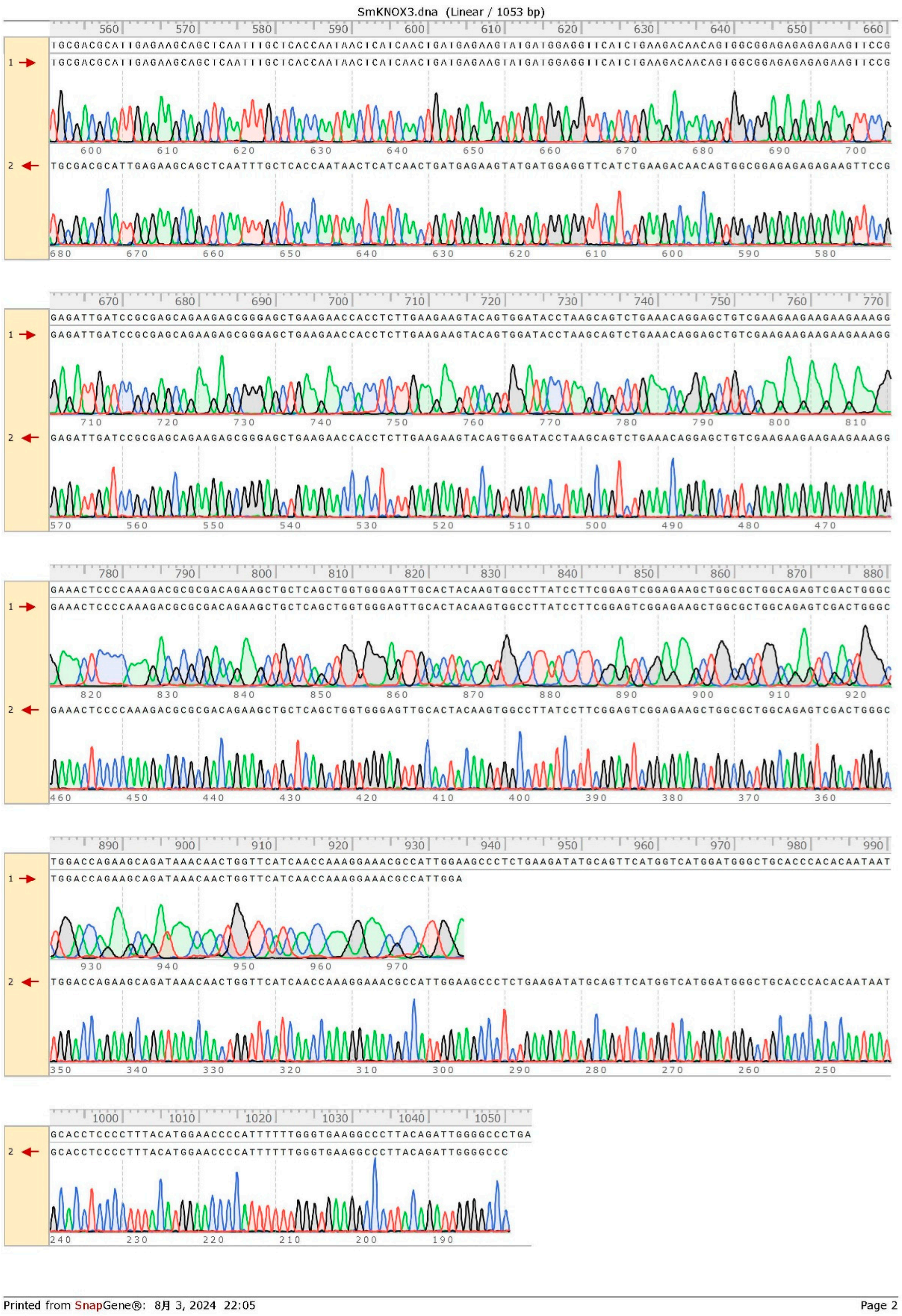

Figure S3. The gene cloning results of SmKNOX3. The base sequence above represents the sequence from reference genome, and the numbers above it represent the base positions. The base sequence below represents the Sanger sequencing result of gene cloning, and the bottom represents the chromatogram of Sanger sequencing.

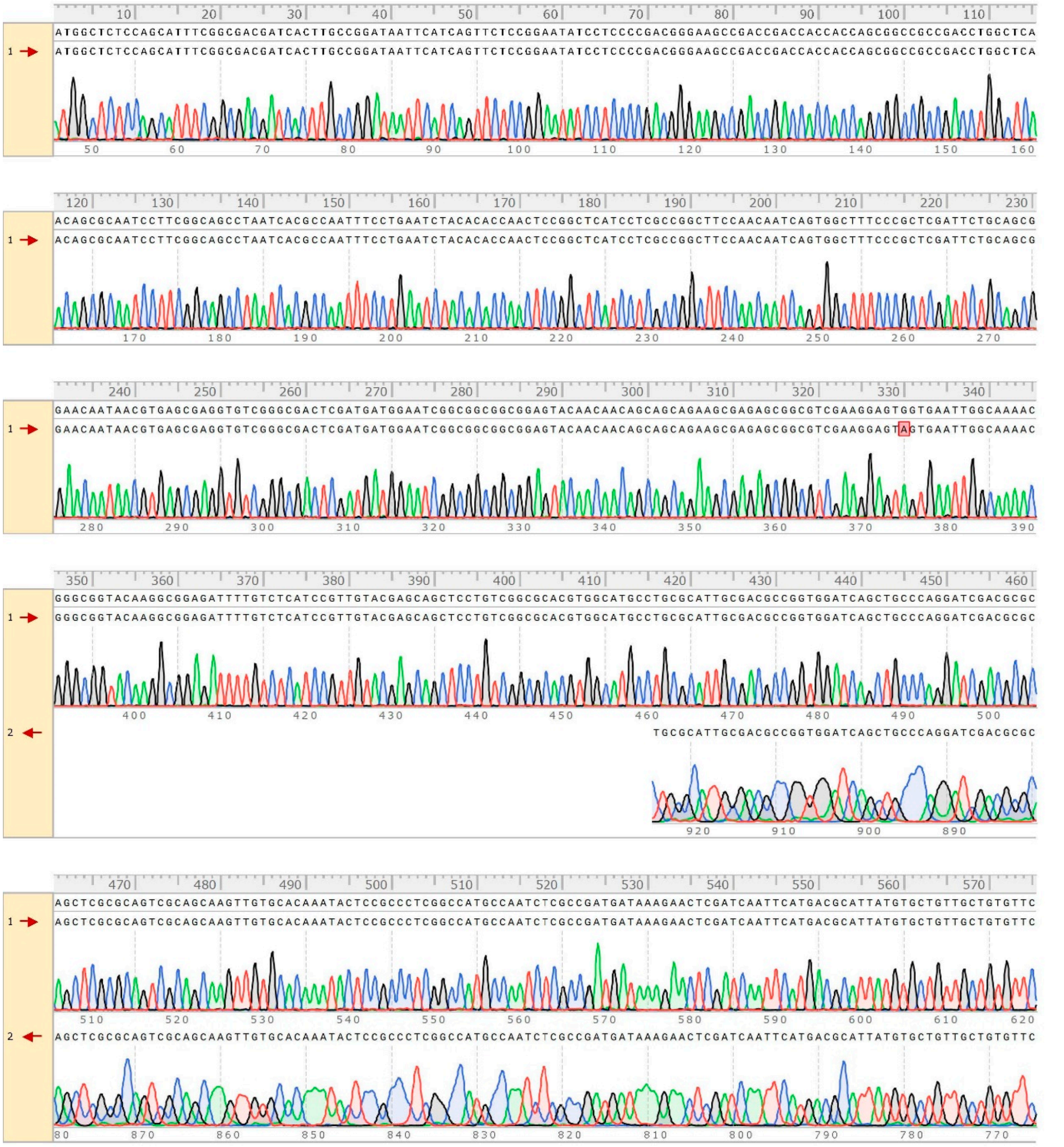
ATGGCTCTCCAGCATTTCGGCGACGATCACTTGCCGGATAATTCATCAGTTCTCCGGAATATCCTCCCCGACGGGAAGCCGACCGACCACCACCAGCGGCCGCCGACCTGGCTCA
1 ATGGCTCTCCAGCATTTCGGCGACGATCACTTGCCGGATAATTCATCAGTTCTCCGGAATATCCTCCCCGACGGGAAGCCGACCGACCACCACCAGCGGCCGCCGACCTGGCTCA
ACAGCGCAATCCTTCGGCAGCCTAATCACGCCAATTTCCTGAATCTACACACCAACTCCGGCTCATCCTCGCCGGCTTCCAACAATCAGTGGCTTTCCCGCTCGATTCTGCAGCG
1 ACAGCGCAATCCTTCGGCAGCCTAATCACGCCAATTTCCTGAATCTACACACCAACTCCGGCTCATCCTCGCCGGCTTCCAACAATCAGTGGCTTTCCCGCTCGATTCTGCAGCG
GAACAATAACGTGAGCGAGGTGTCGGGCGACTCGATGATGGAATCGGCGGCGGCGGAGTACAACAACAGCAGCAGAAGCGAGAGCGGCGTCGAAGGAGTGGTGAATTGGCAAAAC
1 GAACAATAACGTGAGCGAGGTGTCGGGCGACTCGATGATGGAATCGGCGGCGGCGGAGTACAACAACAGCAGCAGAAGCGAGAGCGGCGTCGAAGGAGTAGTGAATTGGCAAAAC
GGGCGGTACAAGGCGGAGATTTTGTCTCATCCGTTGTACGAGCAGCTCCTGTCGGCGCACGTGGCATGCCTGCGCATTGCGACGCCGGTGGATCAGCTGCCCAGGATCGACGCGC
1 GGGCGGTACAAGGCGGAGATTTTGTCTCATCCGTTGTACGAGCAGCTCCTGTCGGCGCACGTGGCATGCCTGCGCATTGCGACGCCGGTGGATCAGCTGCCCAGGATCGACGCGC
2 TGCGCATTGCGACGCCGGTGGATCAGCTGCCCAGGATCGACGCGC
AGCTCGCGCAGTCGCAGCAAGTTGTGCACAAATACTCCGCCCTCGGCCATGCCAATCTCGCCGATGATAAAGAACTCGATCAATTCATGACGCATTATGTGCTGTTGCTGTGTTC
1 AGCTCGCGCAGTCGCAGCAAGTTGTGCACAAATACTCCGCCCTCGGCCATGCCAATCTCGCCGATGATAAAGAACTCGATCAATTCATGACGCATTATGTGCTGTTGCTGTGTTC
2 AGCTCGCGCAGTCGCAGCAAGTTGTGCACAAATACTCCGCCCTCGGCCATGCCAATCTCGCCGATGATAAAGAACTCGATCAATTCATGACGCATTATGTGCTGTTGCTGTGTTC

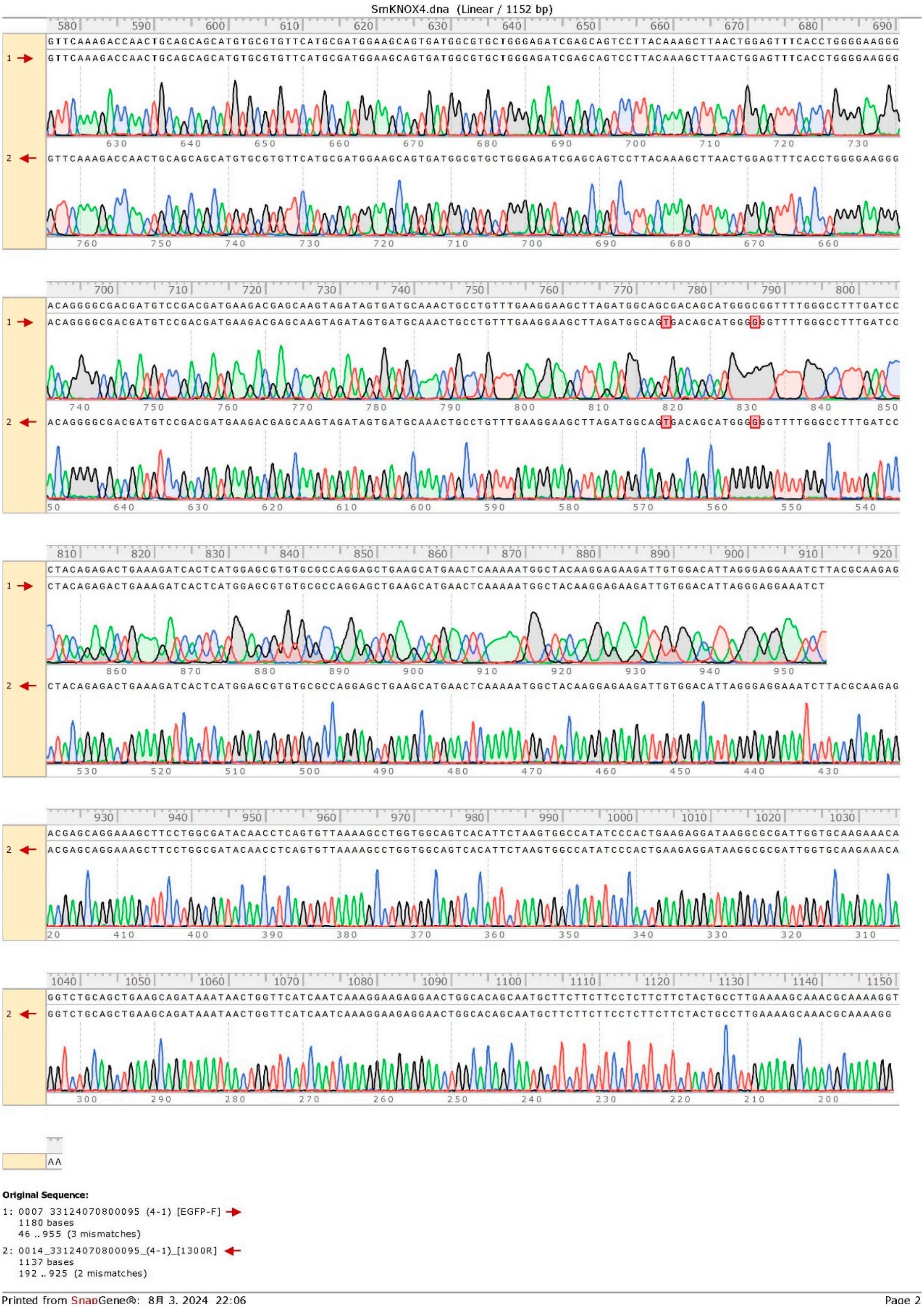

Figure S4. The gene cloning results of SmKNOX4. The base sequence above represents the sequence from reference genome, and the numbers above it represent the base positions. The base sequence below represents the Sanger sequencing result of gene cloning, and the bottom represents the chromatogram of Sanger sequencing.

SmKNOX5.dna (Linear / 783 bp)

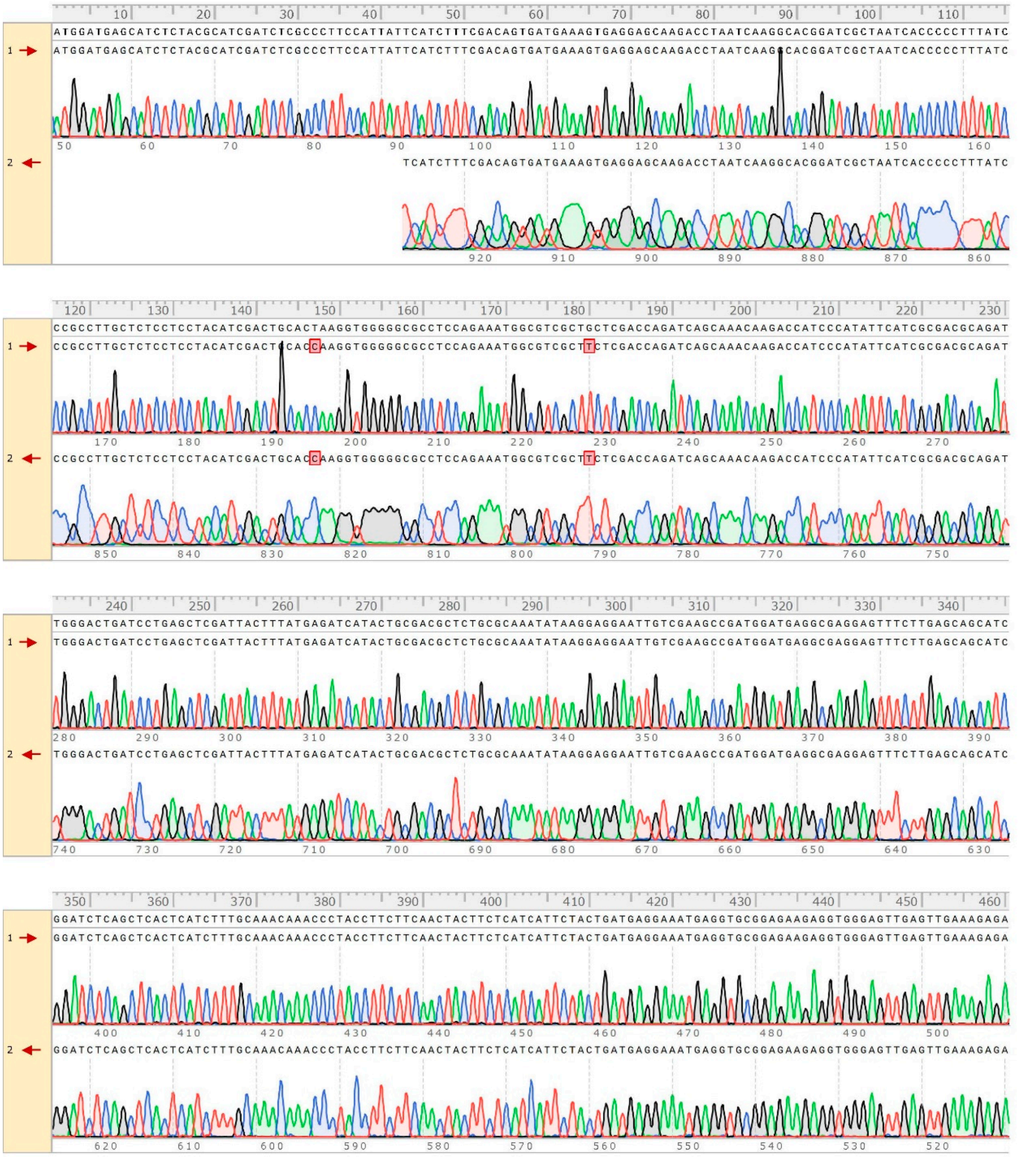

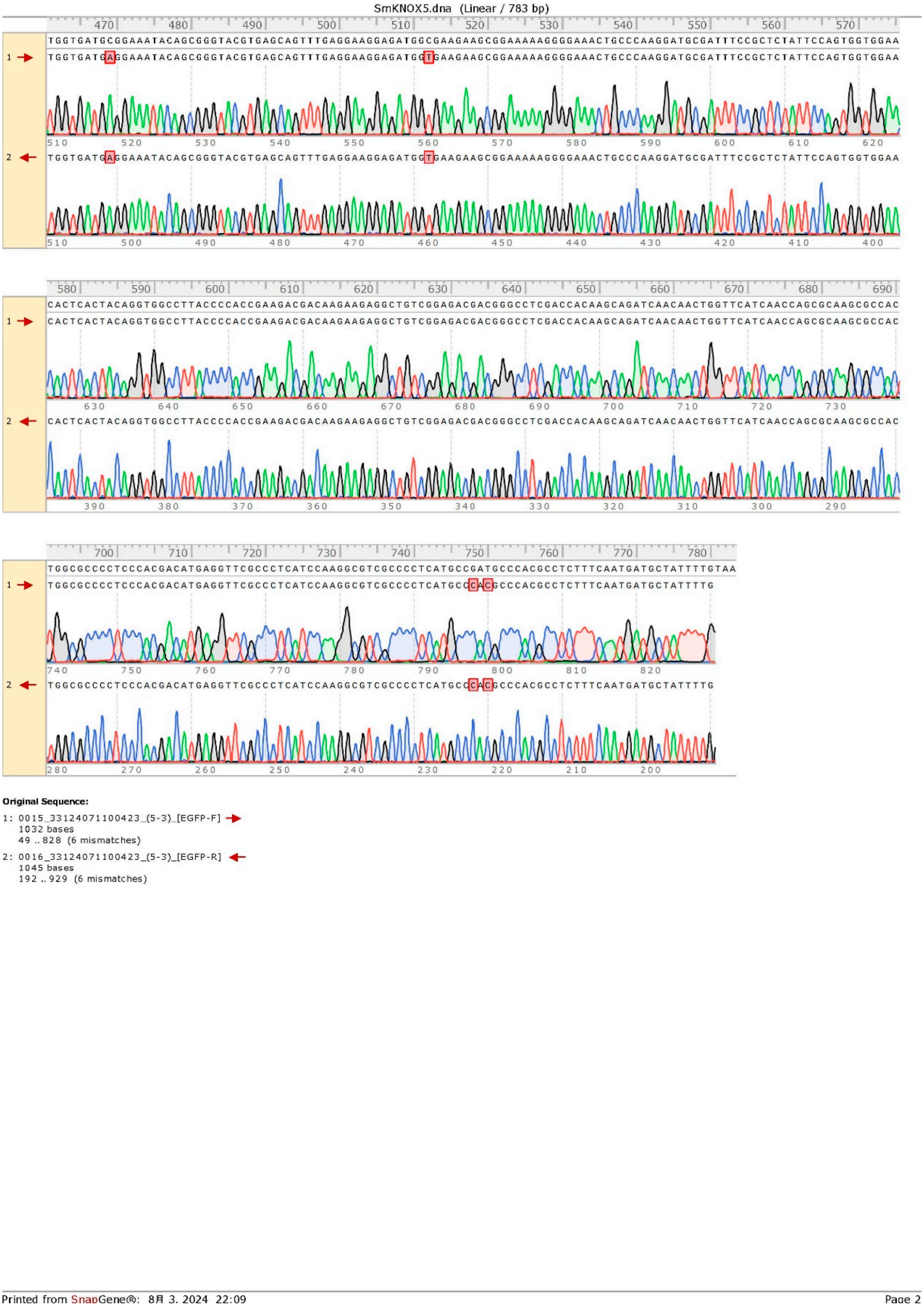

Figure S5. The gene cloning results of SmKNOX5. The base sequence above represents the sequence from reference genome, and the numbers above it represent the base positions. The base sequence below represents the Sanger sequencing result of gene cloning, and the bottom represents the chromatogram of Sanger sequencing.

Sequence: SmKNOX6.dna (Linear / 840 bp)
Enzymes: Unique 6+ Cutters (20 of 653 total) Unique Cutters **Bold**

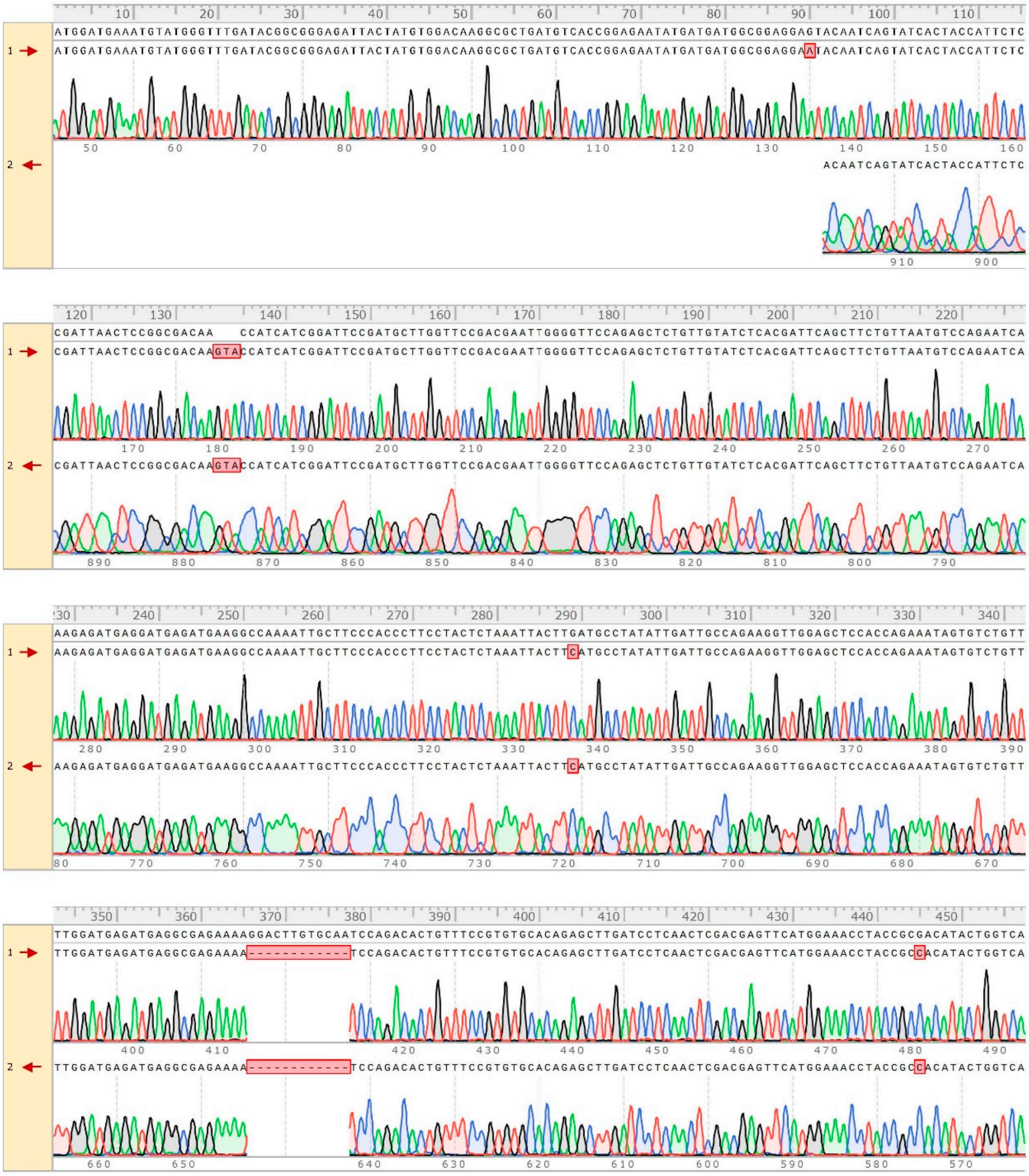

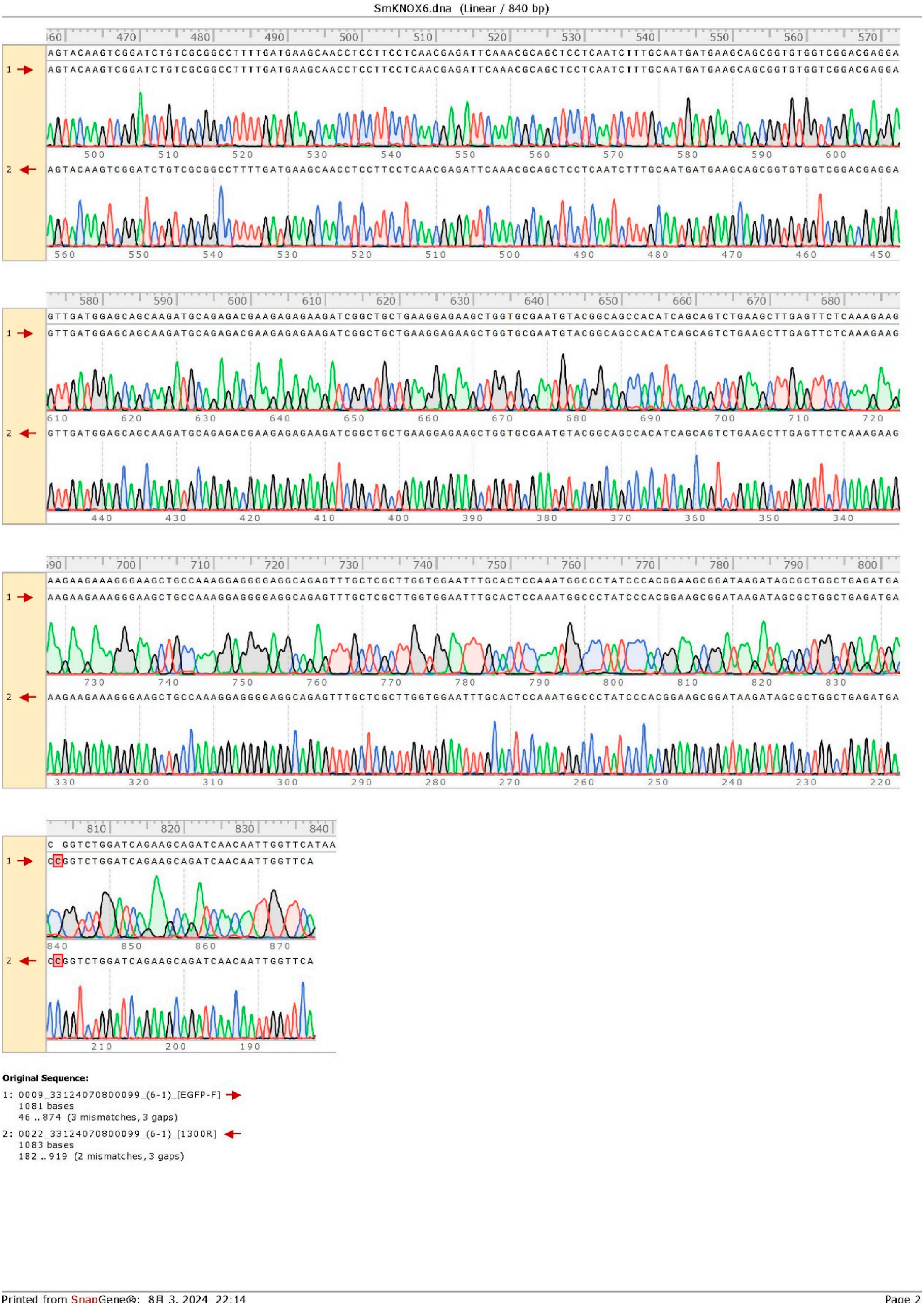

Figure S6. The gene cloning results of SmKNOX6. The base sequence above represents the sequence from reference genome, and the numbers above it represent the base positions. The base sequence below represents the Sanger sequencing result of gene cloning, and the bottom represents the chromatogram of Sanger sequencing.

Sequence: SmKNOX7.dna (Linear / 867 bp)
Enzymes: Unique 6+ Cutters (20 of 653 total) Unique Cutters **Bold**

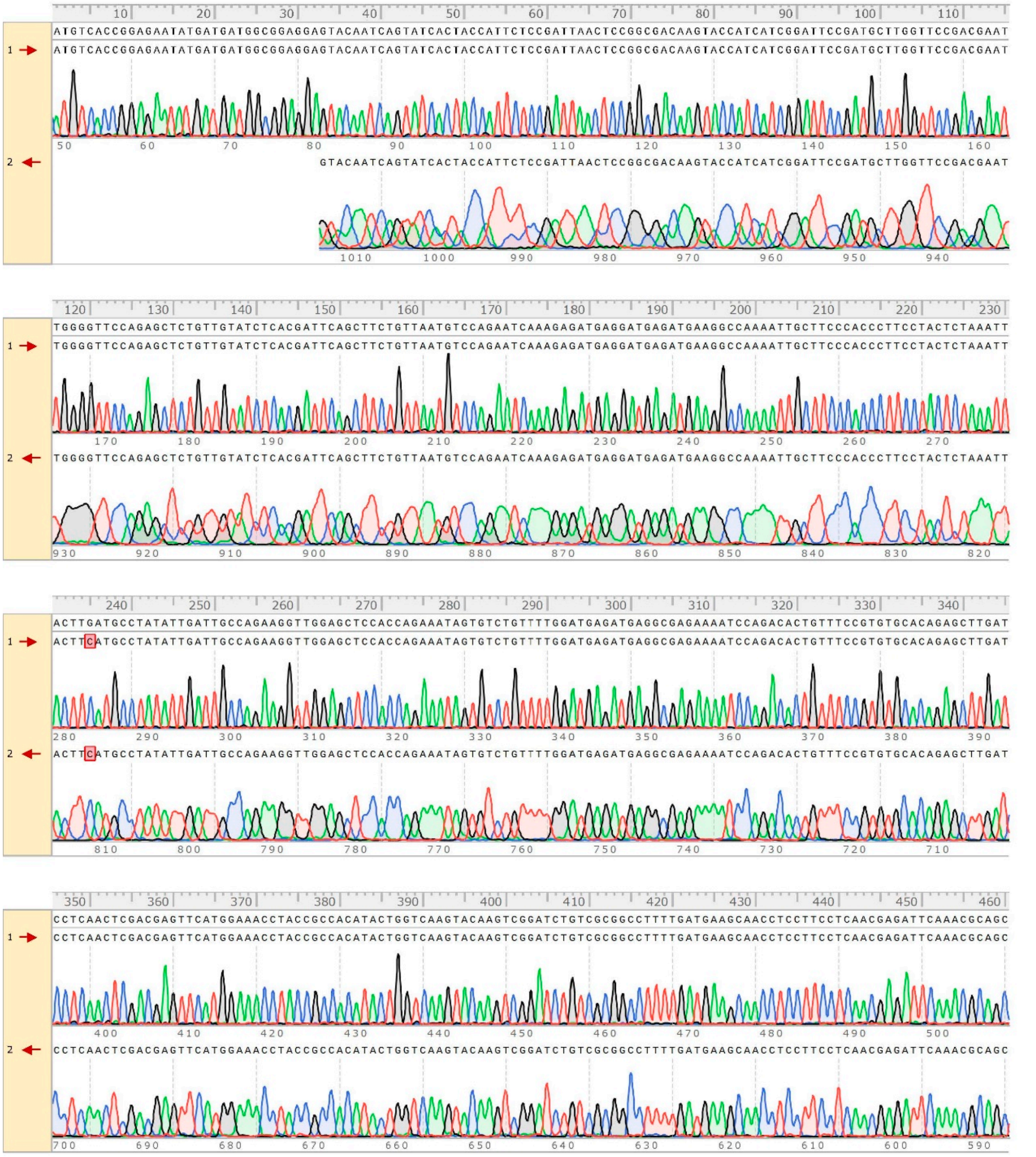

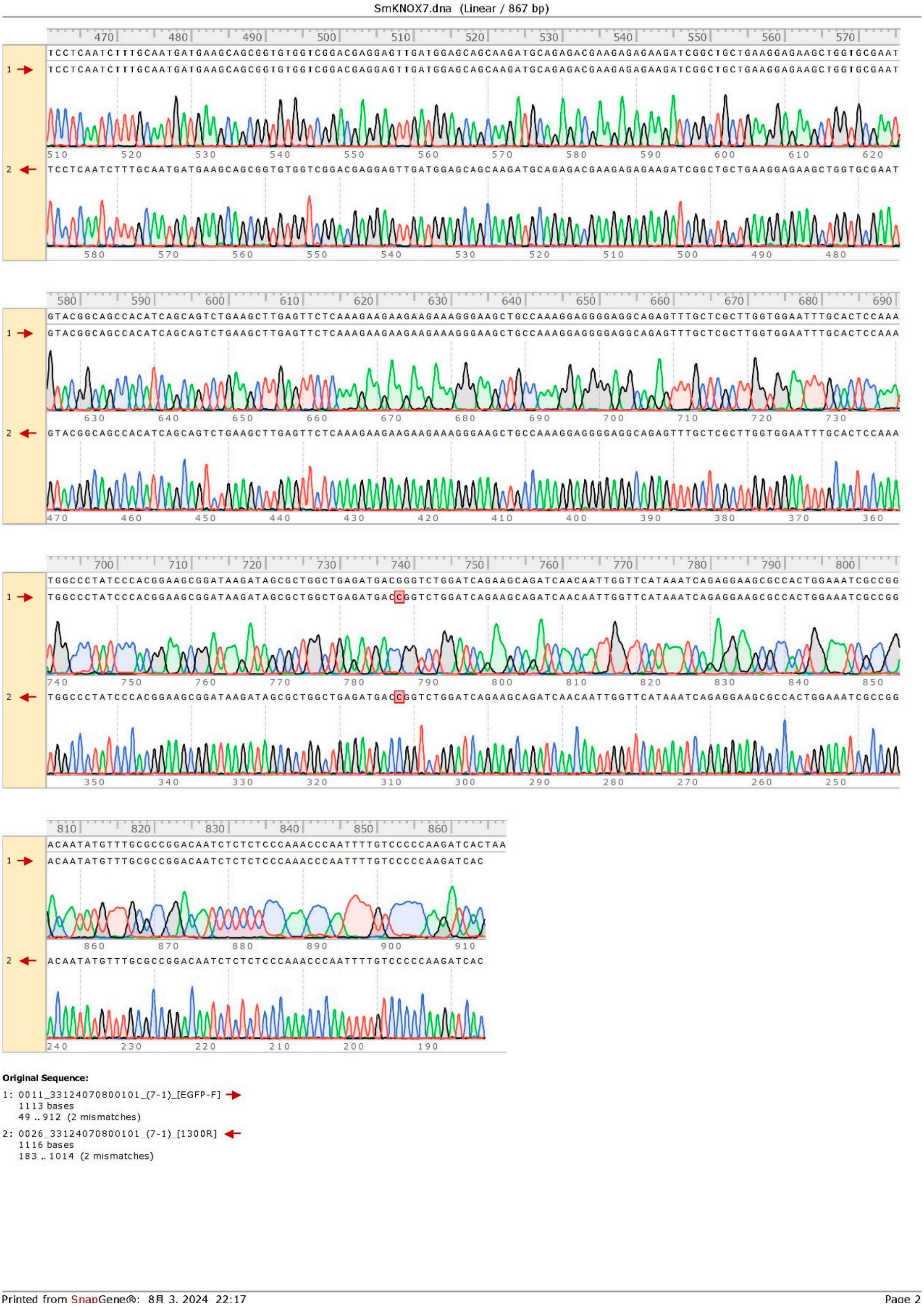

Figure S7. The gene cloning results of SmKNOX7. The base sequence above represents the sequence from reference genome, and the numbers above it represent the base positions. The base sequence below represents the Sanger sequencing result of gene cloning, and the bottom represents the chromatogram of Sanger sequencing.

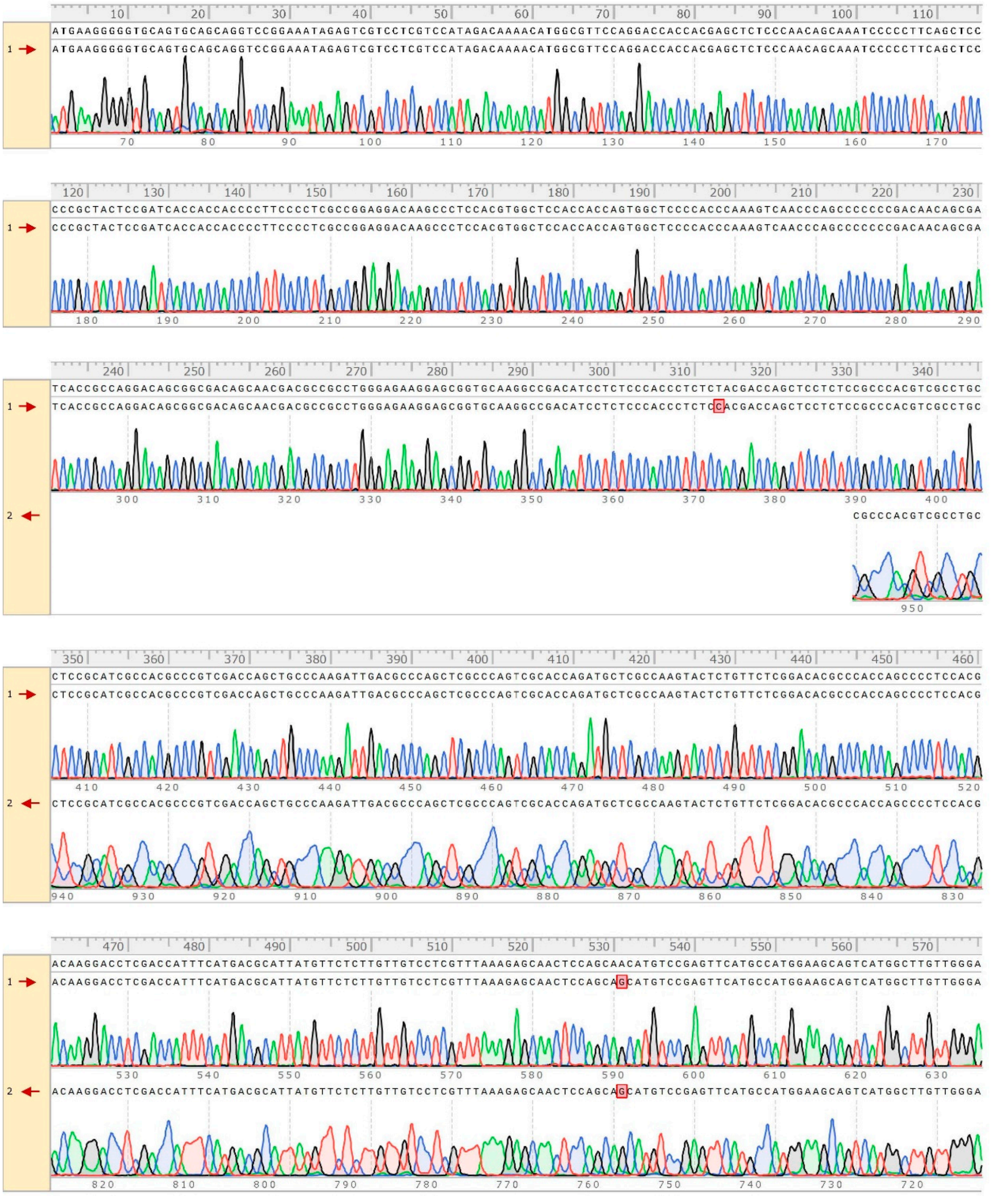
ATGAAGGGGGTGCAGTGCAGCAGGTCCGGAAATAGAGTCGTCCTCGTCCATAGACAAAACATGGCGTTCCAGGACCACCACGAGCTCTCCCAACAGCAAATCCCCCTTCAGCTCC
1 ATGAAGGGGGTGCAGTGCAGCAGGTCCGGAAATAGAGTCGTCCTCGTCCATAGACAAAACATGGCGTTCCAGGACCACCACGAGCTCTCCCAACAGCAAATCCCCCTTCAGCTCC
CCCGCTACTCCGATCACCACCACCCCTTCCCCTCGCCGGAGGACAAGCCCTCCACGTGGCTCCACCACCAGTGGCTCCCCACCCAAAGTCAACCCAGCCCCCCCGACAACAGCGA
1 CCCGCTACTCCGATCACCACCACCCCTTCCCCTCGCCGGAGGACAAGCCCTCCACGTGGCTCCACCACCAGTGGCTCCCCACCCAAAGTCAACCCAGCCCCCCCGACAACAGCGA
TCACCGCCAGGACAGCGGCGACAGCAACGACGCCGCCTGGGAGAAGGAGCGGTGCAAGGCCGACATCCTCTCCCACCCTCTCTACGACCAGCTCCTCTCCGCCCACGTCGCCTGC
1 TCACCGCCAGGACAGCGGCGACAGCAACGACGCCGCCTGGGAGAAGGAGCGGTGCAAGGCCGACATCCTCTCCCACCCTCTCCACGACCAGCTCCTCTCCGCCCACGTCGCCTGC
2 CGCCCACGTCGCCTGC
CTCCGCATCGCCACGCCCGTCGACCAGCTGCCCAAGATTGACGCCCAGCTCGCCCAGTCGCACCAGATGCTCGCCAAGTACTCTGTTCTCGGACACGCCCACCAGCCCCTCCACG
1 CTCCGCATCGCCACGCCCGTCGACCAGCTGCCCAAGATTGACGCCCAGCTCGCCCAGTCGCACCAGATGCTCGCCAAGTACTCTGTTCTCGGACACGCCCACCAGCCCCTCCACG
2 CTCCGCATCGCCACGCCCGTCGACCAGCTGCCCAAGATTGACGCCCAGCTCGCCCAGTCGCACCAGATGCTCGCCAAGTACTCTGTTCTCGGACACGCCCACCAGCCCCTCCACG
ACAAGGACCTCGACCATTTCATGACGCATTATGTTCTCTTGTTGTCCTCGTTTAAAGAGCAACTCCAGCAACATGTCCGAGTTCATGCCATGGAAGCAGTCATGGCTTGTTGGGA
1 ACAAGGACCTCGACCATTTCATGACGCATTATGTTCTCTTGTTGTCCTCGTTTAAAGAGCAACTCCAGCAGCATGTCCGAGTTCATGCCATGGAAGCAGTCATGGCTTGTTGGGA
2 ACAAGGACCTCGACCATTTCATGACGCATTATGTTCTCTTGTTGTCCTCGTTTAAAGAGCAACTCCAGCAGCATGTCCGAGTTCATGCCATGGAAGCAGTCATGGCTTGTTGGGA

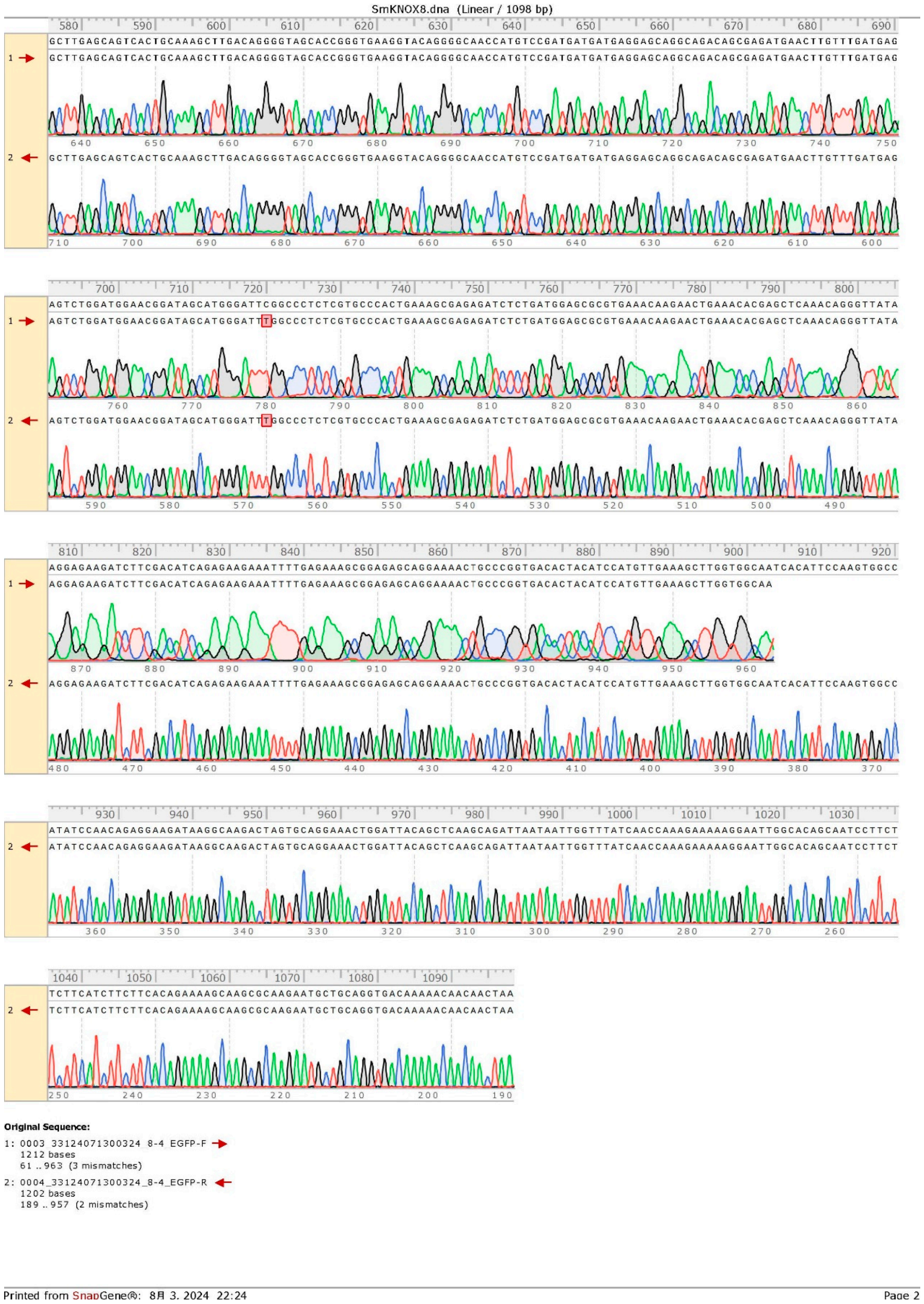

Figure S8. The gene cloning results of SmKNOX8. The base sequence above represents the sequence from reference genome, and the numbers above it represent the base positions. The base sequence below represents the Sanger sequencing result of gene cloning, and the bottom represents the chromatogram of Sanger sequencing.

Sequence: SmKNOX9.dna (Linear / 1125 bp)
Enzymes: Unique 6+ Cutters (32 of 653 total) Unique Cutters **Bold**

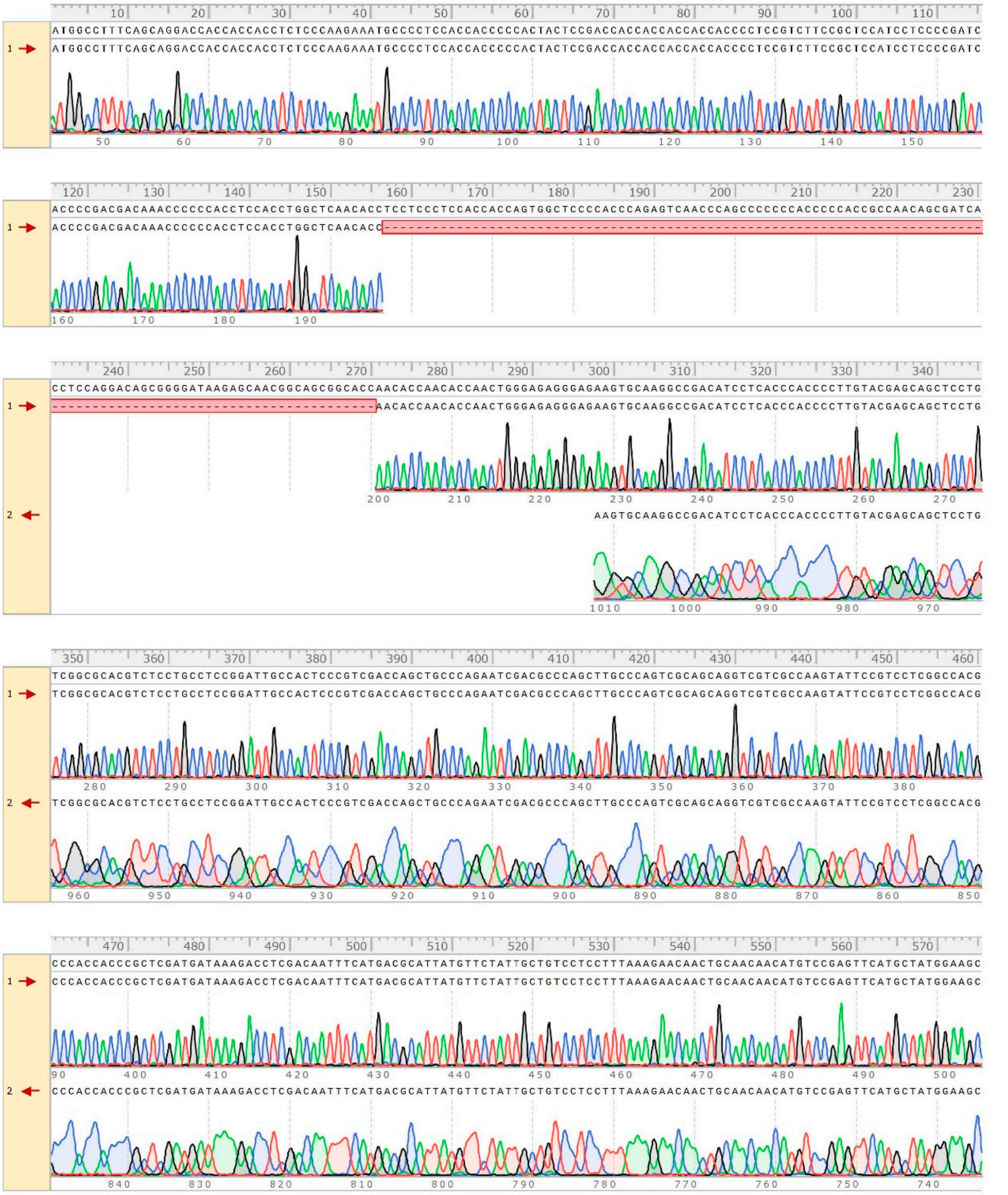

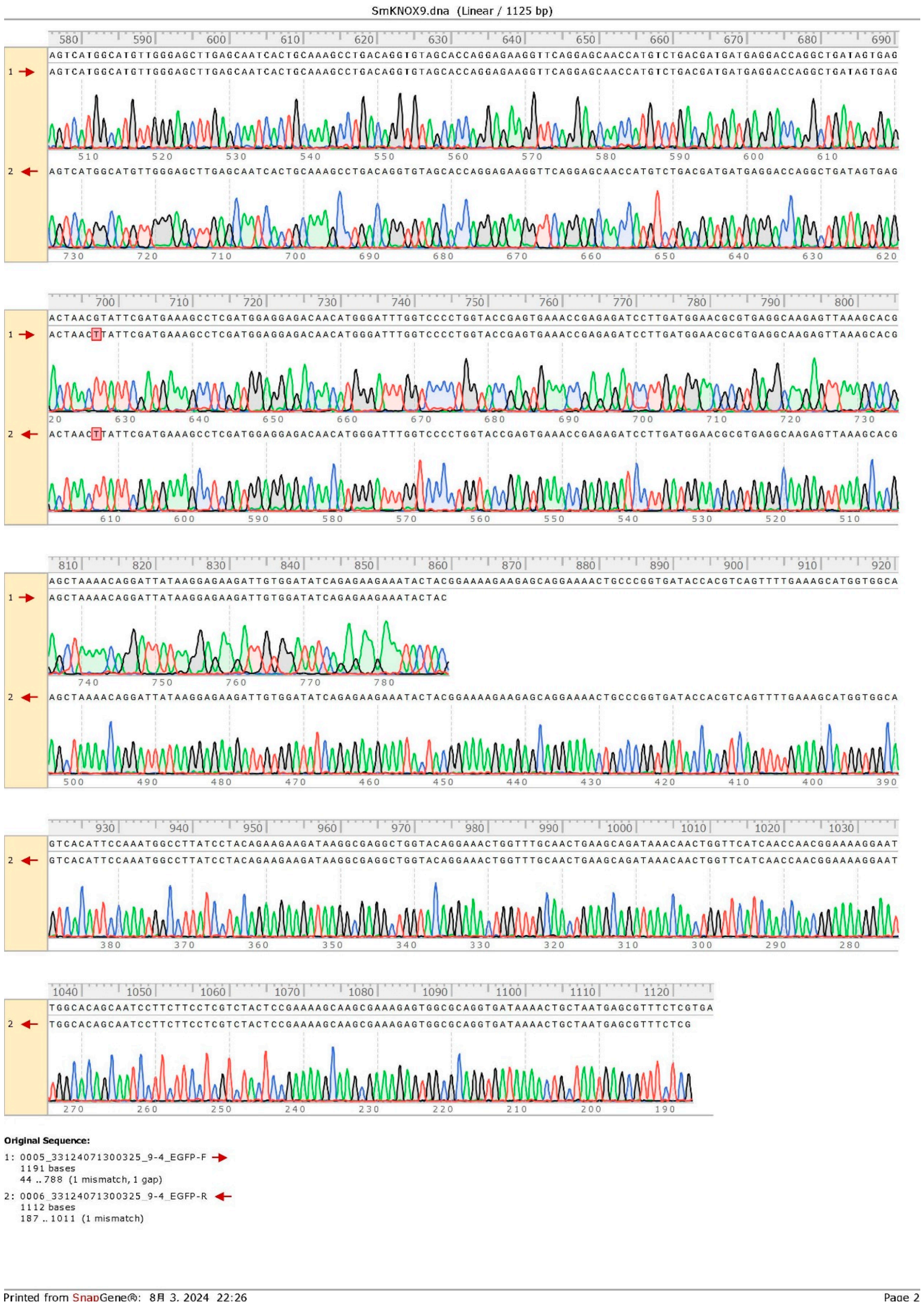

Figure S9. The gene cloning results of SmKNOX9. The base sequence above represents the sequence from reference genome, and the numbers above it represent the base positions. The base sequence below represents the Sanger sequencing result of gene cloning, and the bottom represents the chromatogram of Sanger sequencing.

SmKNOX10.dna (Linear / 957 bp)

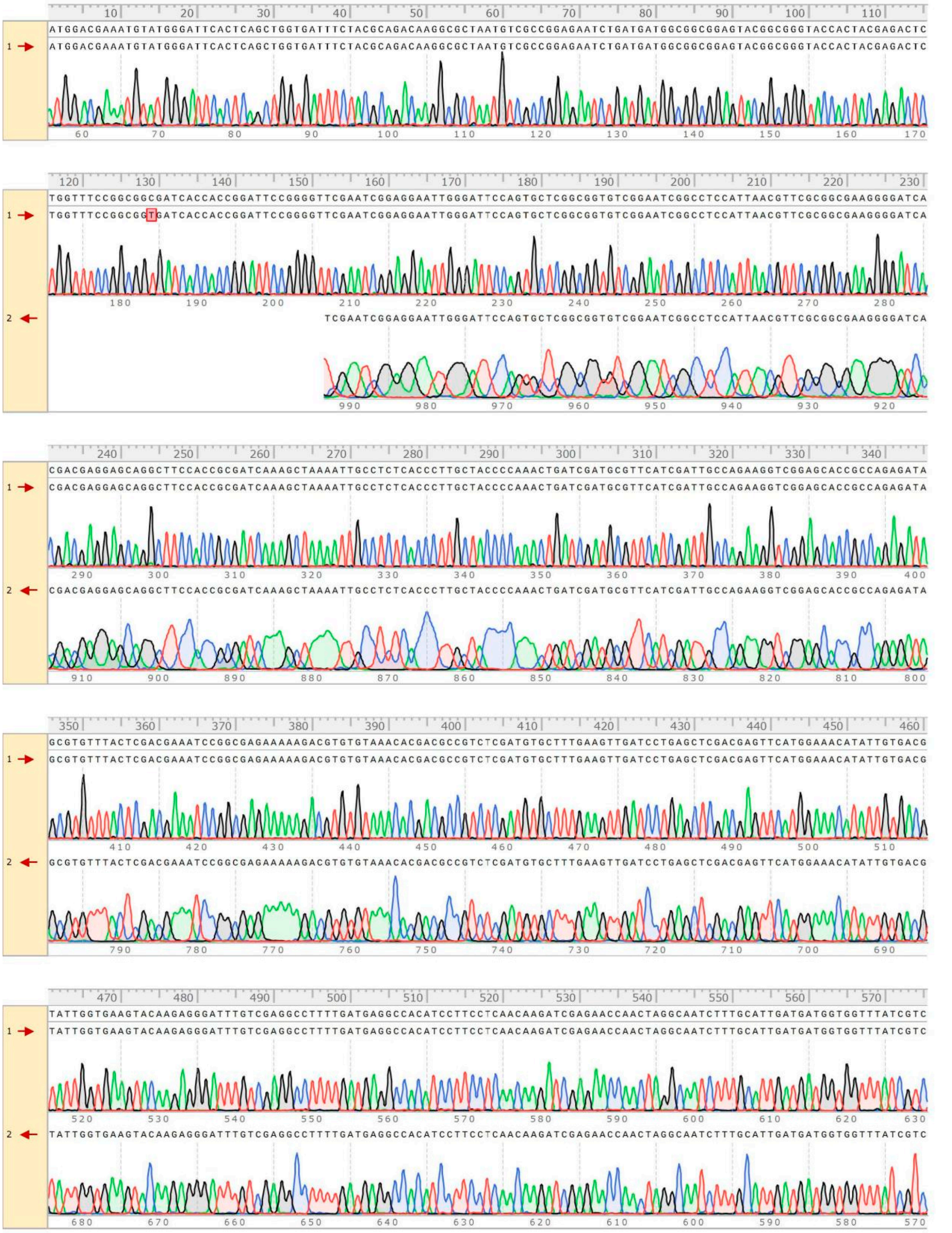

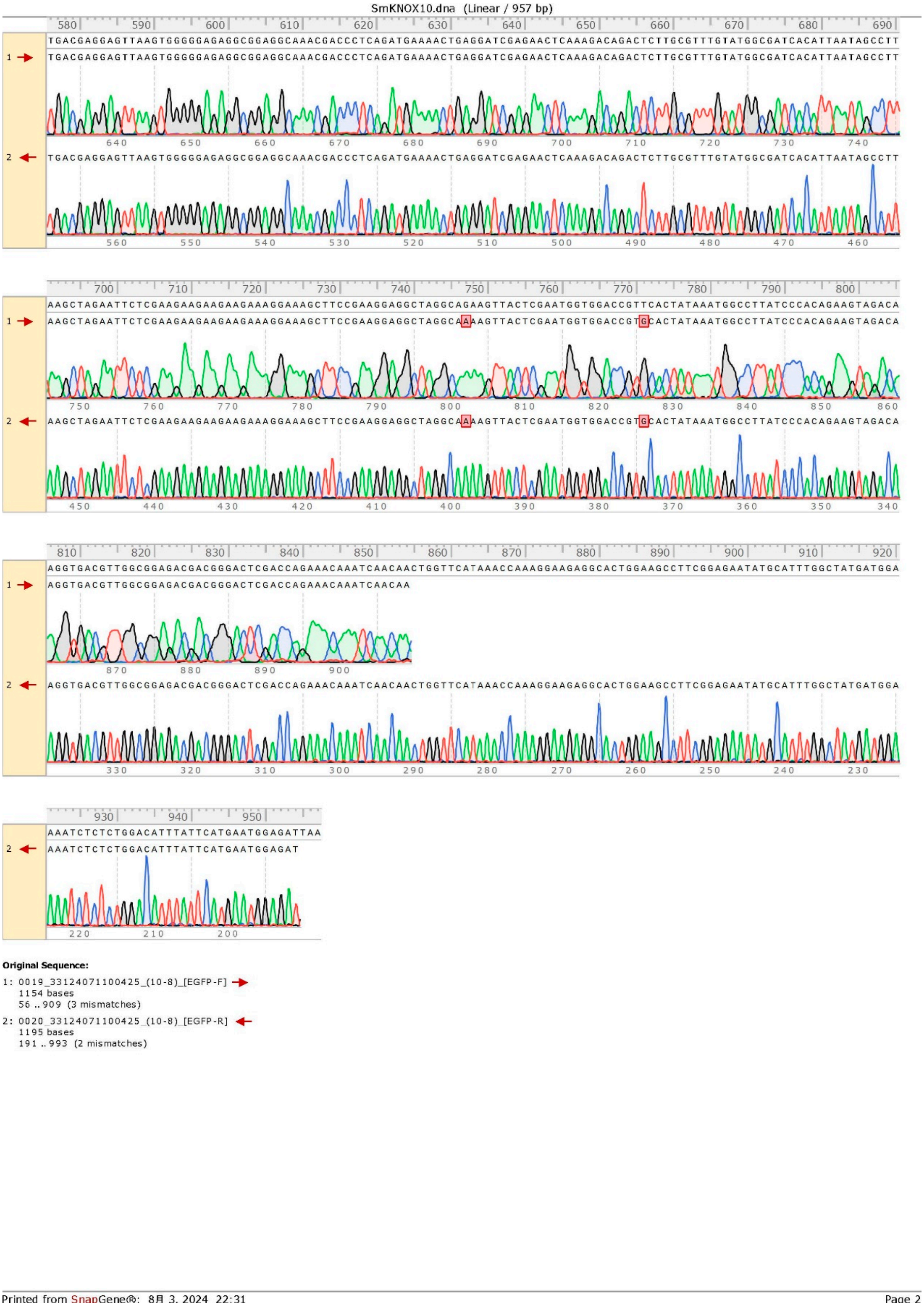

Figure S10. The gene cloning results of SmKNOX10. The base sequence above represents the sequence from reference genome, and the numbers above it represent the base positions. The base sequence below represents the Sanger sequencing result of gene cloning, and the bottom represents the chromatogram of Sanger sequencing.
